# Effects of Small Extracellular Vesicles Derived From Mesenchymal Stromal Cells on Acute Kidney Injury: A Rat Ischemia-Reperfusion Model

**DOI:** 10.7759/cureus.84411

**Published:** 2025-05-19

**Authors:** Shani Zilberman Itskovich, Marina Sova, Ramzia Abu-Hamad, Nour Frage, Shima Esawi, Moshe Stark, Sara Neuman, Lital Sela-Tzuriano, Shimon Slavin, Shai Efrati

**Affiliations:** 1 Nehprology and Hypertension, Shamir Medical Center, Zerifin, Israel; 2 Sackler School of Medicine, Tel Aviv University, Tel Aviv, ISR; 3 Nephrology, Shamir Medical Center, Zerifin, ISR; 4 Biochemistry, Tel Aviv Medical Center, Tel Aviv, ISR; 5 Center for Innovative Cancer Immunotherapy &amp; Cellular Medicine, Biotherapy International, Tel Aviv, ISR

**Keywords:** aki (acute kidney injury), exosomes, msc, placenta, small extracellular vesicles, wharton’s jelly

## Abstract

Extracellular vesicles (EVs) are membrane-bound vesicles, containing nucleic acids, proteins, lipids, amino acids, and metabolites, used for intercellular communication. Several studies indicate that treatment with EVs derived from mesenchymal stromal cells (MSCs) has beneficial effects in acute kidney injury (AKI). MSCs derived from human Wharton’s jelly are relatively non-immunogenic with no MHC class II expression and can serve as a viable source for treating human diseases. The aim of this study was to evaluate the effect of EVs derived from human cord tissue-derived MSCs on ischemic/reperfusion (I/R)-induced AKI.

A rat I/R-induced AKI model was used, and a total of 33 rats were used in the study. Rats underwent unilateral nephrectomies with simultaneous clamping of the contralateral kidney for 60 minutes, followed by reperfusion. The treatment group received human MSC-derived small EVs, from a single donor, or placebo, and after 48 hours, the rats were sacrificed. Blood was used to evaluate renal function and the inflammatory cascade. Kidneys were taken for histopathologic and inflammation examinations.

The EV-treated group significantly improved their renal function compared to the placebo group. Mean creatinine was 1.0 mg/dL in the EV-treated group versus 5.4 mg/dL in the placebo, p=0.005. With respect to the intrarenal inflammatory response, the EV-treated group significantly increased its anti-inflammatory cytokine interleukin-10 levels and attenuated the complement activation compared to the placebo.

Perinatal tissue-derived EVs are potentially beneficial for clinical use since they can overcome many safety concerns related to stem cell use. Further clinical trials are needed to better assess these EVs.

## Introduction

Recent data have highlighted the potential benefits of mesenchymal stromal cells (MSCs) in the prevention of acute kidney injury (AKI) [1]. Many mechanisms have been proposed, but those underlying kidney recovery remain unclear [2,3]. Moreover, MSCs were demonstrated to be beneficial even when being delivered intravenously, with the majority of the injected cells accumulating in the lungs and not reaching the targeted injured kidney [4]. One proposed mechanism by which MSCs exert their remote effects is through the paracrine secretion of extracellular vesicles (EVs), specifically exosomes [5-7]. In a previous study conducted by our group, we demonstrated that MSCs derived from perinatal tissue have an immunomodulatory effect on renal ischemic/reperfusion (I/R) injury. We attributed this effect to the amelioration of the intrarenal complement activation and intrarenal inflammatory responses within 48 hours from treatment initiation [4]. Since the improvement in renal function occurred earlier than would be expected if it were due to intrarenal MSC proliferation and differentiation, it was hypothesized that paracrine signaling was responsible for the observed effects. Consequently, small EVs were identified as potential key players.

EVs are 50-200 nm in diameter, with membrane-bound EVs containing proteins, mRNAs, miRNAs, lipids, amino acids, and metabolites [8]. They are derived from endosomes and facilitate intercellular communication [8]. EVs are secreted into the extracellular space, can be isolated from the culture medium, and can be preserved for future use [9,10]. Several studies show that treatment with MSC-derived EVs can produce similar effects on AKI as MSCs themselves [7]. The mechanisms proposed for the protective effect of EVs on kidney damage include inhibition of apoptosis, immunomodulation activity with complement inhibition, and angiogenesis [5,6]. Thus, MSC-derived EVs might replace whole cell treatment and possibly enable regenerative therapy [9].

EVs can be isolated from MSCs derived from different adult tissues, mainly bone marrow, adipose tissue, and newborn tissues including the placenta and umbilical cord [11]. The beneficial use in AKI was demonstrated mainly in EVs from MSCs derived from bone marrow and also from cord blood, Wharton’s jelly, renal tissue, liver, urine, embryonic tissue, and adipose tissue [12]. The use of perinatal tissue such as placental or embryonic MSCs presents great opportunity for future human clinical trials since they are immunotolerant. Placental and embryonic MSCs, as other perinatal cells, do not exhibit MHC-II, have poor MHC-I expression, and express HLA-G [13,14]. Moreover, these cells are easy to isolate without significant risk to the donor and possess a greater expansion capability and faster growth in vitro [15], which makes them a preferred option for future human clinical trials

The immune system plays a crucial role in I/R-induced AKI, involving both the innate and adaptive immune systems [16]. Previous studies demonstrated that the many beneficial effects of MSCs in AKI models are related to blocking the intrarenal complement activation and intrarenal inflammatory responses [4]. This immunomodulatory effect includes suppression of interleukin (IL)-1, IL-6, and tumor necrosis factor (TNF)-α, together with an increase in M2-macrophages and regulatory T-cells [1]. EVs extracted from MSCs produce similar effects including decreases in TNF-α and IL-1-β [5].

The complement system, part of the innate immune system, plays a role in many renal diseases [17]. The complement system is composed of proteins that act as proteases that are mostly synthesized in the liver [18]. However, recent studies have shown that some of the complement components including C1q and C3 produce locally within the kidney [19]. Overactivation of the complement system may lead to secondary damage during the reperfusion phase after renal I/R injury [4,20]. Inhibiting the overactivation of the complement system can attenuate the expected injury [21]. It should be noted that the potential beneficial effects of the complement system inhibition have not been confirmed yet in clinical studies of AKI due to I/R injury [22], and the inhibition of the complement system is reserved for only a few indications, including atypical hemolytic uremic syndrome and thrombotic microangiopathy [23,24].

The complement system is also related to macrophage activation [25]. Macrophages are antigen-presenting cells that can differentiate to M1 pro-inflammatory and M2 anti-inflammatory cells [26]. Tissue’s resident macrophages can derive from embryonic progenitors or hematopoietic stem cells [27]. Accumulation of interstitial macrophages increases post-ischemia from day 2 to day 8 and then decreases [28].

The aim of the current study was to evaluate the effectiveness of EVs derived from human MSCs in a rat model of renal I/R injury and to assess their impact on the inflammatory cascade. The current study is a continuation of a previous study conducted by our group on the use of MSCs in AKI [4].

This article was previously presented as a meeting abstract at the Israel Nephrology and Hypertension Society Scientific Meeting on March 24, 2022.

## Materials and methods

This study was strictly conducted according to the recommendations of the Guide for the Care and Use of Laboratory Animals of the National Institutes of Health [29]. The protocol was approved by the Committee of Animal Experiment Ethics at Assaf-Harofeh Medical Center (Protocol Number: 29/2018), Zerifin, Israel. Surgeries were performed under inhaled halothane anesthesia, combined with buprenorphine treatment (0.1 mg/kg x 2/day), to alleviate pain. All efforts were made to minimize suffering. A previous study conducted by our group explored the effect of MSCs on AKI [4].

Pilot phase

To define the optimal treatment dose for the study, three doses of EVs were tested. For the I/R + EVs treatment group, doses of 50 µg/mL, 100 µg/mL, and 200 µg/mL were tested (three rats per group). Treatment with 200 µg demonstrated the lowest creatinine and urea levels and was used for the rest of the study. The doses of the EVs tested in the pilot phase were extrapolated from previous studies in the field. The difference between the two groups of 100 and 200 µg was minor and not statistically significant. We therefore chose the higher dose to also check safety.

Ischemia/reperfusion model

Twenty-seven eight-week-old male Sprague-Dawley rats, weighing 250-300 g, were used in the study. As discussed earlier by our group [4], the rats were housed in animal cages at a temperature of 25°C with free access to food and water in our institution’s animal facility. Only male rats were included in the study due to the nature of the abdominal incision surgery and the risk of injury to the female rats’ reproductive system.

The rats were assigned to one of the following groups: (1) unilateral nephrectomy, followed by intravenous (IV) injection of saline 0.9% to the tail vein (control + placebo, three rats); (2) unilateral nephrectomy + I/R in the contralateral kidney, followed by IV injection of saline 0.9% (I/R + placebo, 12 rats); (3) unilateral nephrectomy + I/R in the contralateral kidney, followed by an IV injection of EVs (I/R + EVs, 12 rats). The rats were assigned to groups in a sequential manner. One rat underwent I/R followed by saline injection, and the next rat underwent I/R followed by EV injection. After four rats had undergone I/R, one rat underwent unilateral nephrectomy only. The number of rats chosen for the study was based on previous studies conducted by our group, using the smallest number that achieved reproducible and statistically significance results [4].

Surgical procedure

As described in the previous study conducted by our group [4], the rats were anesthetized with halothane under aseptic conditions. Unilateral nephrectomies were performed using an anterior approach laparotomy after clamping the right renal hilum. I/R was performed using an anterior approach laparotomy and included a unilateral nephrectomy of the right kidney, together with 60 minutes of ischemia by clamping the left renal artery and vein, followed by clamp removal and reperfusion. At the end of the procedure, the rats received IV injections of EVs (200 μg EVs per 1 mL of injection) or 1 mL of 0.9% sodium chloride (saline) as a vehicle control through the tail vein. Once recovered, the rats had free access to food and water.

Mesenchymal stromal cells

Cells used in the present work were from Biotherapy International (RN 513854588), a company that cryopreserves placenta and cord tissue-derived cells. A consent signed by the families of each newborn baby is maintained by the company, approving the use of some MSCs for basic research.

As described in a previous study conducted by our group [4], MSCs were isolated from a human single placenta that was donated after delivery. Placental tissue was excised and used for MSC collection according to research protocols that were prepared in compliance with the International Society for Stem Cell Research (ISSCR) guidelines [30].

The placenta at the cord-placenta junction was repeatedly washed in phosphate-buffered saline (PBS) and cut under a laminal flow hood into small pieces (<0.5 mm^3^) that were further incubated in 30 mL of Dulbecco's modified Eagle medium (DMEM), 1% penicillin and streptomycin and 0.5% collagenase B, and filtered to form a single cell suspension. The cells were maintained in an incubator in 75 cm^2^ culture flasks for 48 hours in DMEM containing 10% fetal bovine serum (Biological Industries), L-glutamine, and 1% penicillin and streptomycin at 37°C, 5% CO_2_. Media were changed every three days until the cell monolayer was around 70-80% confluent.

**Figure 1:**
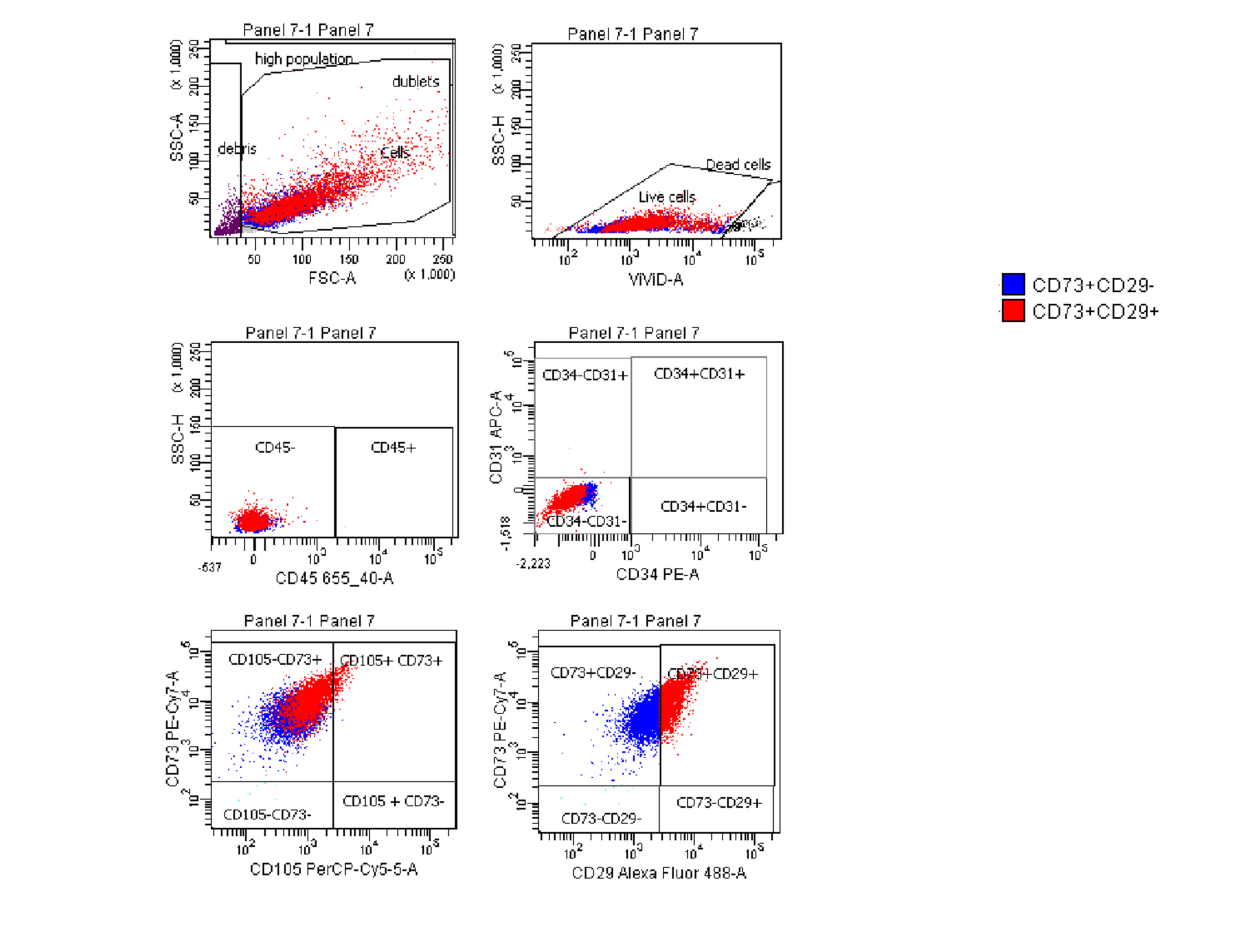
Flow cytometry of mesenchymal stromal cells Flow cytometry characterization of mesenchymal stromal cells used to extract exosomes indicates that cultured cells were negative for leukocyte common antigen CD45, CD34, and CD31 and positive for CD73, CD29, and CD105.

Flow cytometry characterization of the MSCs indicated that cultured cells were positive for CD73 and CD29, with a lower expression of CD105, and were negative for the leukocyte common antigens CD45, CD34, and CD31 (Figure 1).

Isolation of EVs

Media were collected from flasks containing about 2x10^6^ cells, incubated for 48 hours, and centrifuged at 300 X g (10 minutes), and the supernatant was stored at -80ᵒC. EVs were purified from the conditioned medium by differential ultracentrifugation. Larger vesicles were pelleted at 10,000 X g (30 minutes). The supernatant was filtered through a 0.22-mm membrane (Millipore Express plus 0.22-μm PES, 500-mL fast flow), and EVs were pelleted at 100,000 X g (70 minutes using 70-mL polycarbonate bottles and a Type45 Ti rotor (339160; Beckman Coulter, Brea, CA, USA). EV pellets were washed once in 1 mL of sterile PBS and centrifuged for 120 minutes at 100,000 X g in a tabletop ultracentrifuge using a Type45 Ti rotor (339160; Beckman Coulter).

Characterization of EVs: nanoparticle tracking analysis

A NanoSight NS300 (Malvern Panalytical Ltd., Malvern, UK) was used to measure concentration and size distribution of the EVs. Samples were diluted 1:1,000 in PBS and manually injected into the instrument, and videos were acquired at ambient temperature and at camera level 9 for 1 minute per sample (n = 3). EVs were then frozen at -80°C in PBS until further use.

Quantification of EVs

Prior to protein quantification, 50 µL of EVs resuspended in PBS were lysed by adding an equal volume of RIPA buffer (Sigma Aldrich, St. Louis, MO, USA) and cOmplete™, mini, EDTA-free protease inhibitor cocktail (P8340, Sigma Aldrich), followed by incubation at RT for 5 minutes and sonicated for 15 seconds. The insoluble material was pelleted by centrifugation for 15 minutes at 16,000 X g, 4°C. Absorbance was read at 562 nm on a Multiskan SkyHigh plate reader (Thermo Fisher Scientific Inc., Waltham, MA, USA).

Western blot analysis

**Figure 2:**
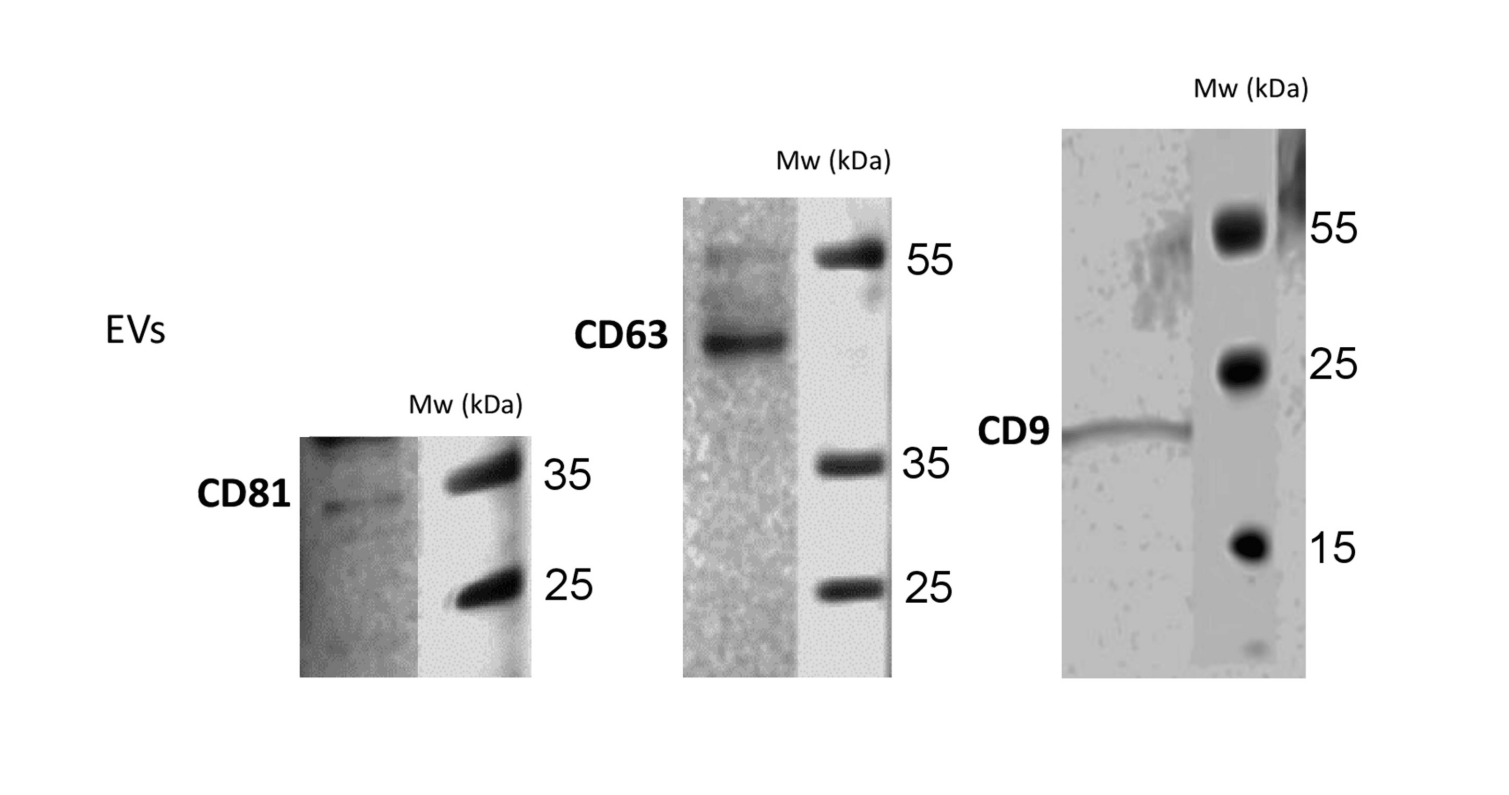
Western blot analysis of exosomes extracted from mesenchymal stromal cells Western blot analysis of exosomes extracted from mesenchymal stromal cells, showing expression of protein markers CD63, CD81, and CD9. EV, extracellular vesicle

Amount of 10 µg of protein was loaded onto 8%-16% Novex WedgeWell tris-glycine gel (XP08162BOX Invitrogen, Thermo Fisher). After transfer to a nitrocellulose membrane (Iblot Mini Stack, IB23002), the membranes were incubated with antibodies and washed, and images were captured using an Odyssey system (Li-Cor, Bad Homburg, Germany) according to the manufacturer’s instructions. Primary antibodies used were CD63 (25682-1-AP) 1:1000, CD81 (66866-1-lg, Proteintech, Rosemont, IL, USA) 1:1000, and CD9 (MAB25292-SP, R&D Systems, Minneapolis, MN, USA) 2 µg/mL (Figure 2). Secondary antibodies used were anti-mouse IgG HRP 7076 and anti-rabbit IgG HRP 7074 (Cell Signaling Technology, Danvers, MA, USA). The protein content of EVs was measured using the Bradford method [31].

Biochemical and immunological evaluations

As described in the previous study conducted by our group [4], the rats were sacrificed under anesthesia with halothane 48 hours following the procedure. Blood was drawn via a cardiac puncture, and the left kidney was removed for pathological evaluation. The blood was centrifuged at 3,000 RPM for 10 minutes, and serum was separated for further tests (Heraeus Megafuge 8R centrifuge, Thermo Scientific). Serum creatinine, urea, and C-reactive protein measurements were performed on a Roche Cobas 701 autoanalyzer (Roche Diagnostics, Mannheim, Germany) according to the manufacturer’s instructions. The complement system, C3 and C4, were assessed by specific ELISAs: rat C3 ELISA (rat: E-25C3, ICL Inc., Portland, OR, USA), rat C4 ELISA (C4 ELISA kit MBS70336, MyBio, Kilkenny, Ireland), and vascular endothelial growth factor (VEGF) (Rat VEGF quantikine ELISA kit, R&D systems) according to the manufacturer’s instructions.

Pathologic and immunofluorescent staining

Following animal sacrifice, kidney tissues were isolated and fixed with 4% paraformaldehyde and subsequently embedded in paraffin. Paraffin-embedded slides were prepared by a standard procedure. The blocks were sectioned at 5-μm-thick slices. One slide from each rat was stained with a hematoxylin-eosin dye for histopathologic examinations under a light microscope. The tissue sections were deparaffinized and rehydrated with a series of xylene, 100%, 90%, 70%, and 50% ethanol prior to immunostaining.

Pathologic evaluation

Computerized morphometry was performed using an Olympus CKX 41 microscope with the CMS-2-M system as part of the Advanced Measurement Systems, Ltd. (Haifa, Israel). The system includes a digital color CCD camera (1,600x1,200 pixels) and a software package for pathology and immunohistochemistry evaluation.

As described in our previous study [4], tubular necrosis was quantified as the percentage of damaged tubules out of the total number of tubules in the examined kidney. Specifically, we calculated this by summing the number of damaged tubules in each specimen and dividing it by the total number of tubules [32]. In addition, nucleus degeneration and proliferation, and protein casts were evaluated and presented as percentage of tubules involved per total tubule count. All pathological evaluation was performed with blinding of the evaluators to the group allocation.

Immunostaining

Antigen retrieval was performed with 10-mM sodium citrate buffer pH 6.0 for 25 minutes in a 95°C water bath. Permeabilization was done with 0.1% Triton X-100 at room temperature for 15 minutes. Tissues were then incubated with blocking buffer (5% mouse or rabbit serum in PBS for 1 hour at room temperature). Slides were then incubated with primary antibodies (IL-1β [MAB501] at 12.5 µg/mL, IL-6 [AF506] at 10 µg/mL, IL-10 [AF519-SP] at 10 µg/mL, TNF-α [NSB2-34372] at 2 µg/mL, C3 [ab48581] at 1:250, and C6 at 1:100 [17239-1-AP]), overnight at 4°C, followed by incubation with secondary antibodies (goat anti-mouse IgG DyLight 488 Cat A90-61602, donkey anti-goat IgG Cat A50-101D3), 1 hour at room temperature. Visualization was done using a Lionheart™ FX automated microscope. Five randomly selected regions of interest (ROIs) per slide were examined, and the areas of interest were interactively selected by two independent observers, blinded to the group allocation, using proper optical threshold and microscope filter combinations. Computer morphometry was performed using the Lionheart FX Automated Live Cell Microscope (BioTek, Winooski, Vermont, USA) at 40X magnification. The system included a digital color camera and software package for immunofluorescence evaluation. The analysis was done using the ROI statistic option of the Gen5 Software. Fluorescence intensity analysis was conducted on the five ROIs from each section. The fluorescence intensity was normalized for cell nuclei (DAPI staining).

Statistical analysis

Data are presented as mean ± standard deviation for continuous data. Differences between groups were evaluated using Mann-Whitney tests when appropriate or a t-test for normal distribution. A p-value of ≤0.05 was assumed to be significant. Data were analyzed using SPSS Version 25.0 (IBM Corp., Armonk, NY, USA).

## Results

I/R-induced AKI

First, we evaluated the effect of I/R injury on renal function to assess the AKI model.

Compared to the control + placebo, I/R + placebo induced acute renal failure, measured by an increase in creatinine levels from 0.9 mg/dL to 5.4 mg/dL and an increase in urea from 122 mg/dL to 407 mg/dL for the control + placebo and I/R + placebo groups (p=0.03 for creatinine and 0.01 for urea). Hemoglobin, white blood cells, and platelets were similar in both groups (Table 1).

**Table 1:** The effect of EV treatment on I/R injury Data are presented as mean ± standard deviation for continuous data. Differences between groups were evaluated using Mann–Whitney/ANOVA tests when appropriate or a t-test for normal distribution. Histological percentage is expressed as percentage of total tubules damaged in the kidney. *p-value < 0.05 for I/R + placebo vs. I/R + EVs groups. **p-value < 0.05 for sham vs. I/R + placebo groups. CD, cluster of differentiation; DAPI, the fluorescence intensity was normalized for cell nuclei; EV, extracellular vesicle; I/R, ischemia/reperfusion; IL, interleukin; SD, standard deviation; TNF, tumor necrosis factor; WBC, white blood cells

	Control + placebo	I/R + placebo	I/R + EVs	p-value for control vs. I/R + placebo	Test statistics	p-value for I/R + placebo vs. I/R + EVs	Test statistics
Hemoglobin (g/dL)	12.3 ± 0.4	12.4 ± 1.3	13.1 ± 0.8	>0.99	U=12	0.32	U=15
WBC (K/µL)	7.4 ± 4	6.2 ± 3	6.2 ± 3.9	0.55	U=8	>0.99	U=24
Platelets (K/µL)	820 ± 15	687 ± 212	747 ± 33	0.09	U=2	0.32	U=15
Systemic renal function evaluation							
Creatinine (mg/dL)	0.9 ± 0.6**	5.4 ± 2.1	1.0 ± 0.5*	0.03	U=1	0.01	U=2
Urea (mg/dL)	122 ± 125**	407 ± 136	118 ± 71*	0.01	U=0	0.01	U=3
Systemic complement markers							
C3 (µg/L)	54.8 ± 7.6	64.6 ± 38	149 ± 72.6*	0.08	U=30	0.01	U=15
C4 (ng/L)	2.55 ± 0.74	8.9 ± 9.5	20 ± 17.2*	0.15	U=30	0.02	U=12
Renal histological/pathology evaluation							
Proliferation (% ± SD)	0 ± 0	10 ± 11	10 ± 6	0.21	U=7	0.18	U=11
Acute tubular necrosis (% ± SD)	0 ± 0**	53 ± 20	30 ± 14*	<0.01	U=0	0.04	U=7
Tubular hyalin casts (% ± SD)	4 ± 9	12 ± 10	5 ± 5	0.08	U=5	0.82	U=16
Intra-renal inflammatory evaluation							
C3 (intensity per DAPI)	13.74 ± 4.38**	32.95 ± 18.8	17.94 ± 7.38*	0.01	U=48	0.01	U=48
C6 (intensity per DAPI)	5.68 ± 5.48**	15.34 ± 3.8	20.02 ± 5.37*	0.01	U=84	0.02	U=84
IL-1β (intensity per DAPI)	5.6 ± 1.2	5.4 ± 2.3	5.8 ± 1.7	0.68	U=299	0.25	U=414
IL-6 (intensity per DAPI)	3.35 ± 1.0	3.5 ± 1.2	3.3 ± 0.9	0.82	U=323	0.68	U=138
IL-10 (intensity per DAPI)	26.4 ± 1.6	24.3 ± 1.4	27.6 ± 9.8*	0.21	U=237	0.01	U=239
TNF-α (intensity per DAPI)	18.8 ± 0.8	20.8 ± 8.1	23.9 ± 8.5	0.80	U=103	0.22	U=249
CD68 (intensity per DAPI)	8.02 ± 7.71**	2.34 ± 0.81	1.46 ± 1.15*	0.01	U=32	0.02	U=32
CD163 (intensity per DAPI)	9.65 ± 3.83**	6.03 ± 3.59	3.27 ± 2.48*	0.01	U=44	0.01	U=44

To conclude, the I/R injury model presented a severe kidney function impairment, without effect on systemic cell count.

EV treatment

Renal Function

The EVs-treated group significantly improved in both creatinine and urea compared to the placebo group. Creatinine was 1.0 mg/dL and 5.4 mg/dL in the I/R + EVs and I/R + placebo treatment groups, respectively (p=0.005). Urea was 118 mg/dL and 407 mg/dL in the I/R + EVs and I/R + placebo treatment groups, respectively (p=0.006) (Table 1).

Renal Pathology

**Figure 3:**
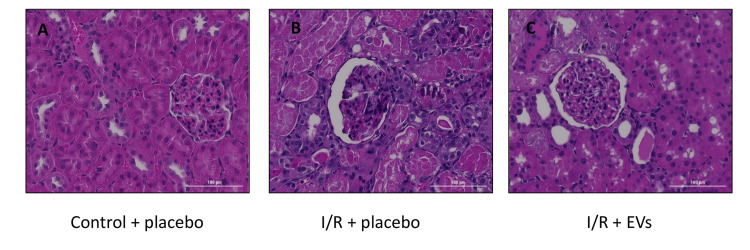
Renal pathology evaluation Hematoxylin-eosin slides of renal pathology (cortex) demonstrating a normal kidney in the control group (A), n=3 rats, severe ATN, and hyalin casts in proximal tubules in the I/R + placebo group (B), n=12 rats, and moderate ATN and hyalin casts in proximal tubules in the I/R + EVs group (C), n=12 rats. ATN, acute tubular necrosis; EV, extracellular vesicle; I/R, ischemia/reperfusion

As detailed in Table 1 and Figure 3, the percentage of acute tubular necrosis (ATN) from total tubules in the kidney was significantly lower in the EVs treatment group (53 ± 20) compared to the I/R + placebo group (30 ± 14), p=0.04.

To conclude, the EVs treatment group presented an ameliorating effect on serum kidney function tests and renal pathological evaluation compared to the placebo group.

Systemic Inflammatory Response

The evaluation of the systemic and local inflammatory response was based on a previous study with MSCs [1]. The results of this study demonstrated that C-reactive protein was relatively low (<0.3 mg/L) and did not change significantly in all study groups (data not shown). There was also no significant change in the white blood cells. VEGF was 60 ± 59 pg/mL in the I/R + placebo group and 26.8 ± 22.7 pg/mL in the I/R + EVs group but did not reach statistical significance (p=0.077, data not shown).

The complement system was evaluated using systemic C3 and C4 levels. C3 was 64.6 ± 38 µg/L and 149 ± 72.6 µg/L in the I/R + placebo and I/R + EVs groups, respectively (p=0.009). C4 was 8.9 ± 9.5 ng/L and 20 ± 17.2 ng/L in the I/R + placebo and I/R + EVs treatment groups, respectively (p=0.024) (Table 1).

Renal Immunohistochemical Results

**Figure 4:**
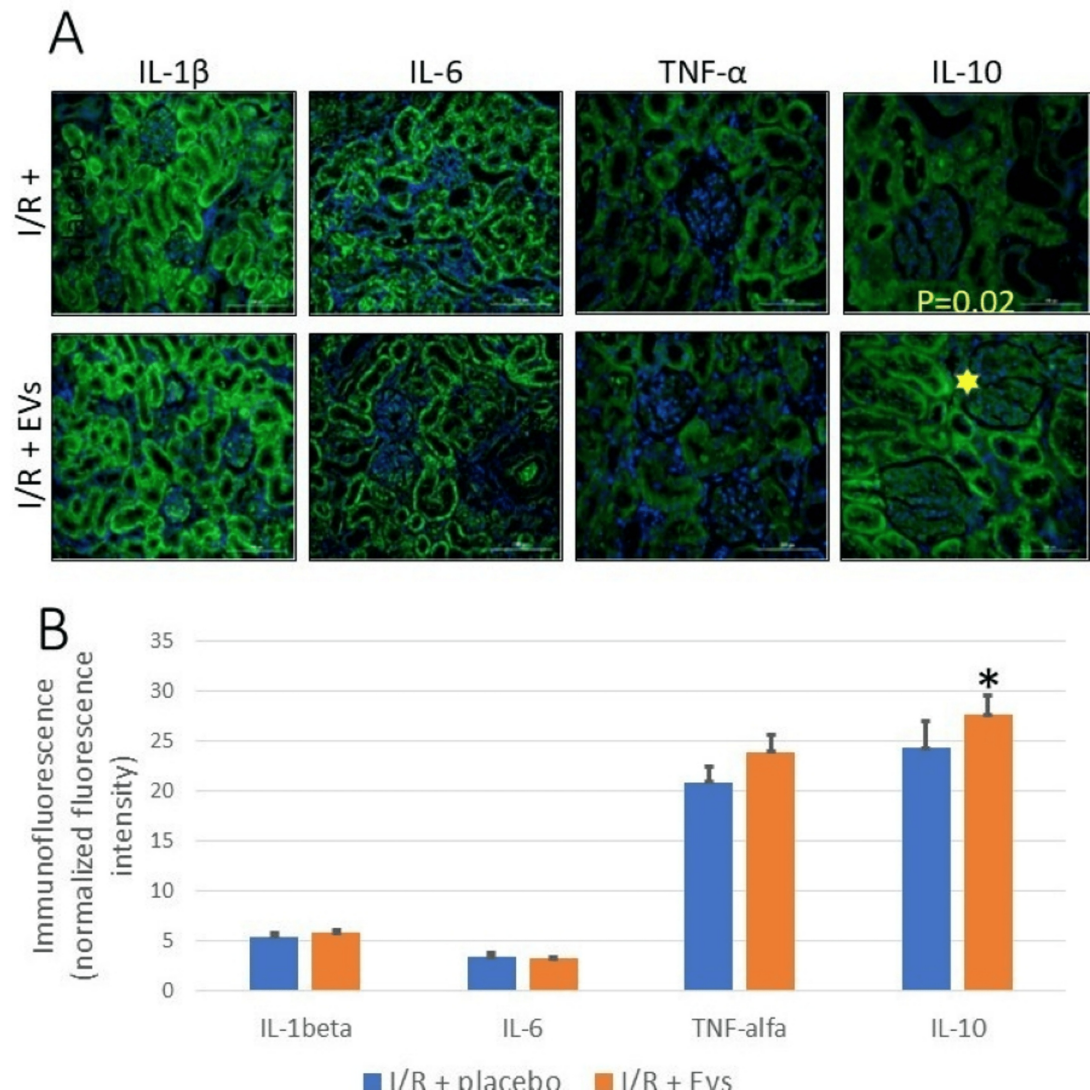
Tissue cytokines A. Intrarenal (cortex) immunofluorescence results of four different cytokines tested in this current study: IL-1β, IL-6, TNF-α, and IL-10. The figures show the intensity of immunofluorescence between the I/R + placebo and I/R + EVs treatment groups. Only IL-10 shows a statistically significant increase in intensity in the EVs treatment group, while no statistically difference was observed in IL-1β, IL-6, and TNF-α between I/R + placebo and I/R + EVs groups. B. Graphical results of cytokines tested in the current study. *Statistically significant differences. EV, extracellular vesicle; IL, interleukin; I/R, ischemia/reperfusion; TNF, tumor necrosis factor

Compared to the I/R + placebo group, EV treatment significantly increased intrarenal levels of IL-10 (Figures 3, 4). IL-6, IL-1β, and TNF-α were similar in the I/R + EVs and I/R + placebo groups (Table 1 and Figure 4).

Complement System in the Renal Tissue

**Figure 5:**
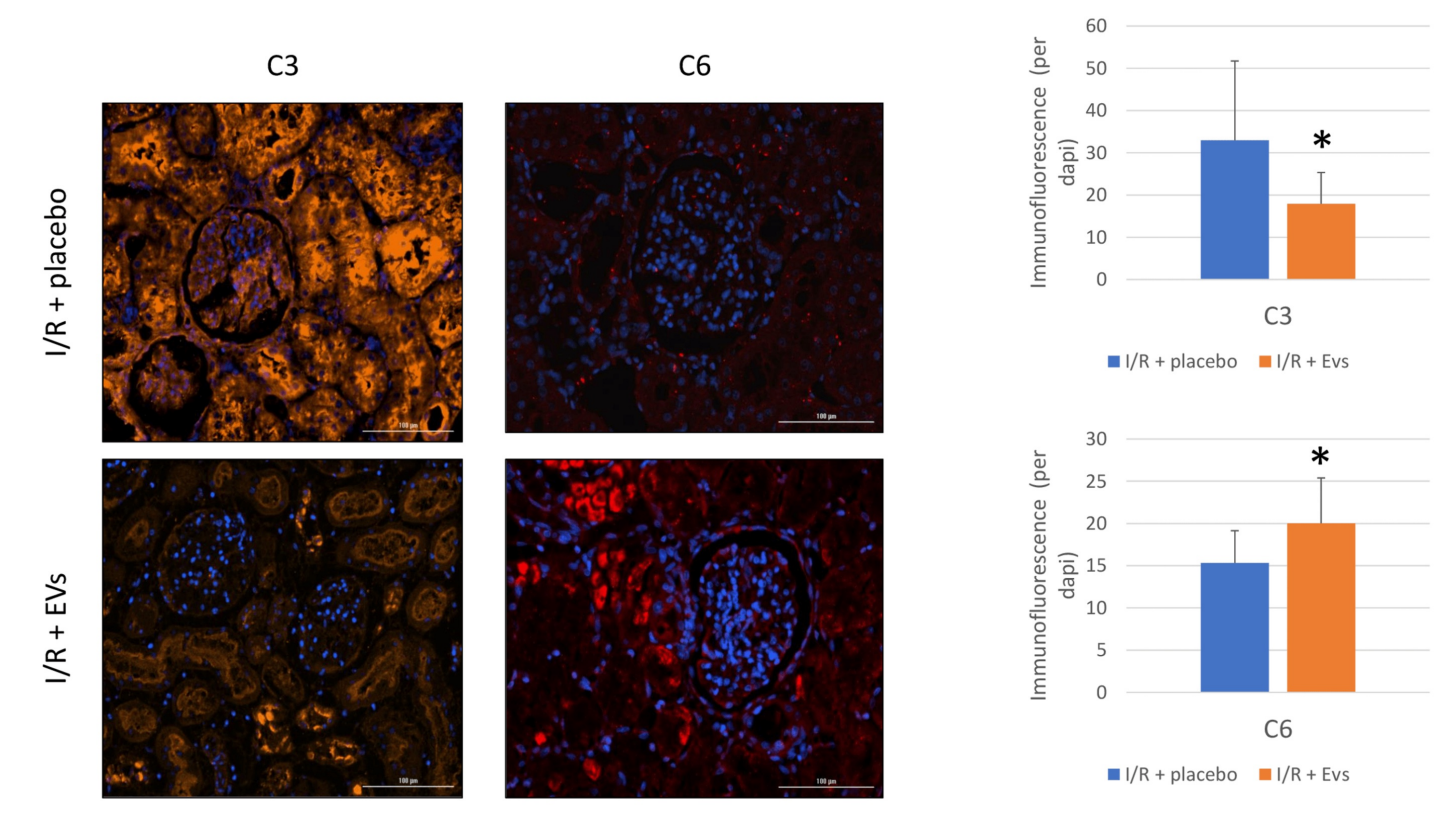
Complement system C3 and C6 levels Intrarenal (cortex) C3 and C6 intensity of immunofluorescence staining, comparing the I/R + placebo and I/R + EVs treatment groups, showing a statistically significant decrease in intrarenal C3 levels and increase in C6 following EVs treatment. Visualization was done by an automated microscope to quantify immunofluorescence per cell nuclei (DAPI). EV, extracellular vesicle; I/R, ischemia/reperfusion

Treatment with EVs ameliorated the expected increase in C3 levels within the renal tissue, as compared to I/R + placebo. Conversely, C6 levels within the renal tissue were higher in the EV-treated group (Figure 5).

Macrophage Levels in the Renal Tissue

**Figure 6:**
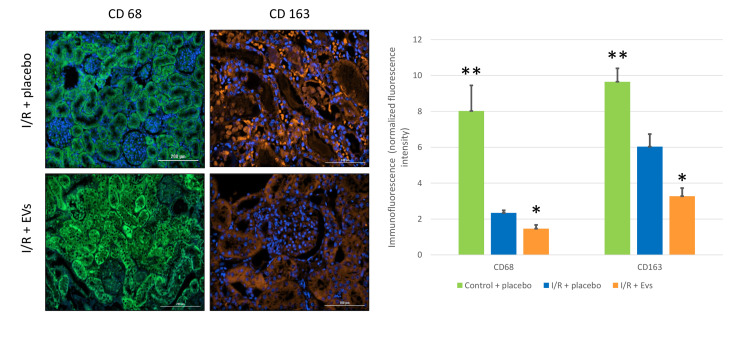
Tissue macrophage populations The intensity of macrophage CD68 and CD163 levels in immunofluorescent staining, following I/R injury and administration of EVs and placebo treatment, presenting a decrease in intensity of both intrarenal CD63 and CD68 levels. Visualization was done by an automated microscope to quantify immunofluorescence per cell nuclei (DAPI). *p-value < 0.05 for I/R + placebo vs. I/R + EVs groups. **p-value < 0.05 for sham vs. I/R + placebo groups. CD, cluster of differentiation; EV, extracellular vesicle; I/R, ischemia/reperfusion

Quantification of macrophages (CD68 and CD163 positive cells) in the tissue demonstrated a significant decrease of macrophages following EVs administration (Figure 6).

To conclude, EV treatment had a significant effect on the complement system, with increase in serum C3 and C4, and intrarenal decrease in C3, which means that less intrarenal consumption may be responsible for the increase in the serum levels. Serum VEGF appeared to be affected but did not reach statistical significance. Intrarenal IL-10 was increased in the EVs treatment group. Intrarenal macrophages were also attenuated, with decrease in macrophage invasion, with both CD68 and CD163 presenting macrophages.

## Discussion

In this current study, we investigated the effects of EVs derived from MSCs sourced from human Wharton’s jelly on kidney function in a rat I/R AKI model. We hypothesized that EV treatment would prevent kidney injury by attenuating the immune system response. The results demonstrate that EV treatment can ameliorate the expected kidney injury by alleviating the inflammatory response, increasing intrarenal IL-10, reducing macrophage accumulation, and ameliorating the complement system activation.

Cumulative preclinical research from various models of acute organ injuries, such as the lungs, liver, heart, and kidneys, supports the potential beneficial use of treatment with MSCs or their secretory nanoparticles [7,33]. In the current study, the focus was the use of EVs from perinatal tissue on the recuperation from AKI. Similar to previous findings, these microvesicles attenuated the inflammatory response and prevented the devastating renal damage at the reperfusion phase [10,34]. The time from the injury to treatment is of significant importance, as can be learned from the clinical study conducted by Swaminathan et al., in which they evaluated the effect of renal function following cardiac surgery [22]. In that study, MSCs were administered relatively late, after the renal injury was established, measured by an increase in creatinine up to 48 hours post-operatively. In this study, the MSCs were shown to be ineffective [22]. Thus, it seems that administration of MSCs or EVs should be done as early as possible while the renal injury is evolving, as the mechanism is immunomodulation and AKI prevention rather than AKI treatment [1,35]. Further studies are needed to define the window of opportunities in which the treatment is beneficial.

In the present study, C3 and C6 staining within the kidney was increased following I/R, but only C3 was attenuated using EV treatment, while C6 remained high, and systemic C3 and C4 increased in the serum. C3 is the first common pathway of the complement system, and after cleavage, C3a serves as an anaphylatoxin and chemotoxin, important for the recruitment of the immune cells, while C3b activates the membrane attack complex (MAC) that contains C5b, C6, C7, C8, and C9 [36,37]. The current study shows that EV treatment reduced intrarenal C3 and does not influence the systemic complement system. The complement system behaves uniquely in different inflammatory contexts. In cases such as autoimmune diseases, there are often low circulating levels of C3/4 during the acute phase of the disease due to their consumption at the tissue level [38]. This phenomenon is particularly evident in conditions such as lupus nephritis and C3 glomerulopathy [18,39]. The increase in C3 levels in our study may indeed signify a resolution of the inflammatory flare-up, as suggested by Breda et al. [38]. The systemic increase in complement proteins could be attributed to heightened production in the liver following acute ischemia [17,18,40], accompanied by a reduction in complement consumption at the tissue level [17], which is associated with renal damage and tubular cell death [4,21]. The increase in C6 within renal tissue did not align with intrarenal C3 levels. C6 is a component of the MAC, which is typically activated through the cleavage of C3, leading to the cleavage of C5 and subsequent initiation of the MAC. However, C6 can also be activated through alternative mechanisms despite lower C3 levels. These include non-conventional pathways such as C5 activation by thrombin in response to acidosis, which may occur during ischemia, or through the use of the C4b2a complex without C3 cleavage [41-43]. Another possibility is that C6, being a mosaic protein composed of several homology modules found in many other proteins, was strongly stained due to the I/R process, which causes cell lysis and presentation of epitopes, but was not activated or cleaved within the tissue to generate C7 and subsequently the cytolytic activity of C9 [42]. Further isolated cellular culture studies are needed to assess this hypothesis.

In our study following I/R injury, the total number of macrophages decreased, which is different from previous studies. This might be explained by differences in warm ischemia time and time period from I/R injury to kidney biopsy tissue. In our study, the ischemia time was longer than in most previous studies in the field (30-45 minutes), while the time to kidney tissue biopsy was relatively short [27,28]. This combination might have led to the death of resident macrophages, while infiltration of bloodstream monocytes had not yet commenced. Further reduction of macrophages was observed following EV treatment. Both CD68- and CD163-presenting cells were attenuated. CD68 is a pan-macrophage marker, and CD163 is a specific M2 marker [44]. Macrophages express C3a receptors on their surface, and mice deficient of complement C3 have reduced macrophage infiltration capabilities [25]. Therefore, the reduction in macrophages could be attributed to the amelioration of complement C3 within the tissue, cellular death resulting from prolonged ischemia time, and the relatively short duration before biopsy, which may not have allowed sufficient time for the infiltration of monocytes from the bloodstream. This phenomenon aligns with observations of diminished macrophage invasion in other porcine models treated with EVs and is associated with the mitigation of immune potential destruction in AKI [45].

An important part of the inflammatory response is regaining tissue homeostasis after an acute injury. This role is carried out by IL-10, which serves as an attenuator and an inflammation regulator [46]. IL-10 could downregulate the systemic or local inflammatory response caused by ischemic AKI [47]. In the current study, IL-10 levels increased after EV treatment, while other cytokines remained unchanged. In an animal model of chronic kidney disease (CKD), treatment with IL-10, delivered as a hydrogel, attenuates macrophage infiltration, apoptosis, and renal fibrosis [48]. IL-10 might induce differentiation of macrophages into M2 macrophages needed for regeneration [49]. The regenerative anti-fibrotic effect of M2 is reported in a later phase of AKI and was first observed 72 hours following I/R injury [50].

In our study, systemic VEGF level appeared to be attenuated following EV treatment but did not reach statistical significance. VEGF is an endogenous angiogenic cytokine with prominent roles in microvascular proliferation and repair, which also maintains vascular network in the kidneys [51]. The beneficial effect of VEGF is mostly related to the recovery phase of the injury and not due to the increase in production during the first hours following I/R injury [52]. In the recovery phase, few weeks following the acute insult, VEGF promotes regeneration and neovascularization [53]. During the early post-insult stage VEGF, 48 hours after the injury as evaluated in the current study, higher VEGF levels may reflect a more severe tissue damage rather than a repair mechanism. The downside of VEGF reduction is that VEGF plays a crucial role in tissue recovery, and lower levels are associated with CKD and renovascular disease [54].

The EVs used in this study were extracted from mesenchymal MSCs isolated from a single placenta's perinatal tissue. Human placenta-derived MSCs have already been used in human clinical trials for several indications such as multiple sclerosis, amyotrophic lateral sclerosis, other neuroinflammatory and neurodegenerative disorders, lupus nephritis, treatment of intractable acute graft versus host disease, kidney transplantation, and sepsis [13,14]. The characteristics of these MSC cells, among other unique properties, make them immune-tolerant and prevent the stimulation of the host immune system against them [13,14]. However, there are still many concerns regarding MSC administration of pluripotent cells [55]. In addition to the benefits of using MSCs, EVs lack the differential potential of MSCs and are easier to preserve [9,10]. Therefore, with respect to quality assurance and long-term safety, Wharton’s jelly MSCs derived from perinatal tissue are better candidates for clinical use. Other origins of MSCs, such as bone marrow, cord blood, and renal tissue, were previously described as beneficial in AKI models, with verifying efficiency and good safety profile. Nevertheless, one of the limitations of the current study is that cells used for the extraction of EVs were all donated from a single placenta. Therefore, more studies are needed with other cell origins in order to ensure that the results presented in this study are consistent.

Previous studies using EVs extracted from MSCs evaluated the immune response to renal damage including IL-10 and macrophages. EVs extracted from bone marrow-derived MSCs showed an increase in IL-10 with attenuation of macrophages as well as IL-6 in a ureteric obstruction model [56]. Microvesicles derived from adipose-derived MSCs showed a decrease in IL-1β and increase in IL-10 in a renal chronic hypoxia model [57]. EVs extracted from umbilical cord-derived MSCs have attenuated TNF-α, IL-6, and IL-1β levels, and increased IL-10 level in a model of chronic renal failure following I/R injury [58]. Our study further advances the understanding of how MSC-derived EVs impact renal I/R injury. It builds on the concept that immune system modulation using EVs can prevent the potential destructive effects of inflammation on the kidneys, as suggested in previous studies, such as those by Lu et al. involving bone marrow-derived MSCs [56]. In contrast, our study utilized EVs extracted from MSCs sourced from Wharton’s jelly in the placenta of a consenting mother immediately after birth. Before moving to clinical trials for AKI, it is crucial to assess various MSC sources for optimal EV extraction. Additionally, our research aims to further evaluate potential measurable biological effects of these EVs. This evaluation is essential to identify reliable biomarkers that provide deeper insights into the biological impacts following administration.

The primary objective of this study was to assess the short-term effects of EV-based treatments on kidney recovery after severe AKI. Without recovery, patients risk progressing to CKD, which is associated with increased mortality, cardiovascular risk, and other comorbidities [59]. While the safety and delayed side effects of mesenchymal stem cell therapy were not the main focus of this study, further research is planned to explore these aspects. However, as discussed, the long-term potential risks are primarily associated with stem cell therapies. Therefore, non-cellular therapies, such as EV treatment, are anticipated to have a safer long-term profile [60,61]. Previous studies on renal disease models, which monitored effects from one day to two weeks post-injection, reported no significant safety concerns. Notably, one study on I/R injury in rats extended the follow-up to six months to evaluate the incidence of CKD post-EV therapy [7]. Despite these findings, there is still a need for more comprehensive studies focusing on long-term safety assessments.

## Conclusions

In conclusion, this study sheds additional light on the mechanism of action related to the use of MSCs and EVs in the setting of AKI. The beneficial effects can be attributed to immune modulation generated by increasing intrarenal IL-10, attenuation of the complement system activation, and macrophage accumulation. Since EVs are only vesicles and not cells, they alleviate many of the safety concerns related to the use of MSCs in clinical settings. Further laboratory studies are needed to evaluate the long-term effects on renal function, and additional clinical trials are necessary to assess their potential beneficial use in humans.
